# Prediction of the Vickers Microhardness and Ultimate Tensile Strength of AA5754 H111 Friction Stir Welding Butt Joints Using Artificial Neural Network

**DOI:** 10.3390/ma9110915

**Published:** 2016-11-10

**Authors:** Luigi Alberto Ciro De Filippis, Livia Maria Serio, Francesco Facchini, Giovanni Mummolo, Antonio Domenico Ludovico

**Affiliations:** Department of Mechanics Mathematics and Management (DMMM), Polytechnic of Bari, Bari 70126, Italy; luigi.defilippis@poliba.it (L.A.C.D.F.); francesco.facchini@poliba.it (F.F.); giovanni.mummolo@poliba.it (G.M.); antoniodomenico.ludovico@poliba.it (A.D.L.)

**Keywords:** Artificial Neural Network (ANN), modeling, simulation, Friction Stir Welding (FSW), mechanical properties, Aluminum Alloy (AA), Ultimate Tensile Strength (UTS), hardness, Heat Effected Zone (HAZ)

## Abstract

A simulation model was developed for the monitoring, controlling and optimization of the Friction Stir Welding (FSW) process. This approach, using the FSW technique, allows identifying the correlation between the process parameters (input variable) and the mechanical properties (output responses) of the welded AA5754 H111 aluminum plates. The optimization of technological parameters is a basic requirement for increasing the seam quality, since it promotes a stable and defect-free process. Both the tool rotation and the travel speed, the position of the samples extracted from the weld bead and the thermal data, detected with thermographic techniques for on-line control of the joints, were varied to build the experimental plans. The quality of joints was evaluated through destructive and non-destructive tests (visual tests, macro graphic analysis, tensile tests, indentation Vickers hardness tests and t thermographic controls). The simulation model was based on the adoption of the Artificial Neural Networks (ANNs) characterized by back-propagation learning algorithm with different types of architecture, which were able to predict with good reliability the FSW process parameters for the welding of the AA5754 H111 aluminum plates in Butt-Joint configuration.

## 1. Introduction

**Figure 1:**
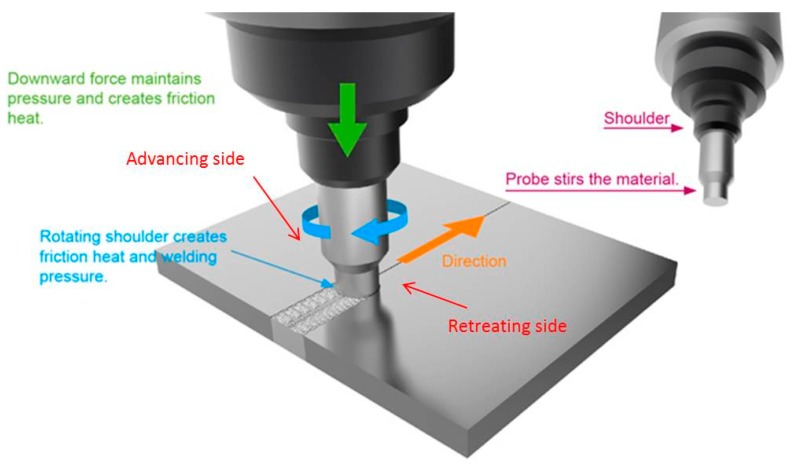
Schematic diagram of the FSW process.

The process of Friction Stir Welding (FSW) is a solid-state welding method based on frictional and stirring phenomena, which was discovered and patented by the Welding Institute of Cambridge in 1991 and documented in the literature by Thomas [1], Nandau et al. [2], and Rodrigues et al. [3]. In this process, welding heat is produced by a rotating non-consumable tool which plunges into the work piece and moves forward. Therefore, the welding is possible thanks to the action of a tool that generates heat by friction between its shoulder and the base material, giving rise to plastic deformation with its pin. Significant advantages can be obtained when it is compared with fusion joining processes for aluminum due to a very low welding temperature: mechanical distortion is practically eliminated, with minimal Heat Affected Zone (HAZ), and there is an excellent surface finish [2]. Additionally, FSW yields no crack formation and porosity right after bonding because of the low input of total heat. The technology relies on the use of a particular tool, as shown in Figure 1, which provides both the necessary heat to the plasticization of the material and the useful motion to scramble the plasticized material and to generate the junction.

In the literature, there are numerous contributions regarding the application of this process, which is used to weld successfully low-melting temperature alloys, steel plates with considerable thickness and dissimilar materials, which are hardly welded with fusion welding processes. Since the FSW process has been discovered, the demand of assembled lightweight metal structures increased.

FSW has been widely used in aerospace, aeronautics, marine sector, fuel tanks and saving food industry for about a decade. It is used to bond aluminum alloys as well as Cu alloys, Ti alloys and steel [4,5]. In particular, Al alloys, thanks to their high strength-to-weight ratio, low density, forming properties, low costs and recyclability, are the most widely employed materials in many automotive, marine and aerospace applications. In this case, the FSW process avoids the tendency of showing cracks and porosity of the aluminum alloy, which frequently occurs after fusion welding.

An aluminum alloy of large aeronautical and automotive interest is the AA5754 H111; however, the FSW process of non-heat-treatable aluminum-magnesium (Al-Mg) alloys (5xxx series) is substantially less explored in the literature. The mechanical properties of the welds produced from Aluminum 5xxx alloys depend mainly on the grain size and the dislocation density, due to the phenomena of plastic deformation and recrystallization, occurring during the FSW process, as shown by Senkara et al. [6] and by Miles et al. [7]. Jin et al. [8] studied the FSW of AA5754 using constant parameters and they have examined the microstructural development and micro hardness distribution in the welds. In the work of Kulekci et al. [9], the effects of the tool pin diameter and the tool rotation speed at a constant travel speed were investigated on fatigue properties of friction stir overlap welded AA5754. Other papers [10,11] provide information on the influence of process parameters on the tensile and on the fatigue behavior of a Friction Stir Welded joint under a single FSW parameter in a tailor-welded blank of AA5754. Nevertheless, these studies were not explicit with regard to the process parameters that were employed. Furthermore, as part of the process control techniques, they cannot provide information about the performance of process during welding and require lengthy testing times, making them feasible for the industrial field. Other authors suggested Infrared Thermography (IRT) to study the thermal behavior of welded joints. Even in this case, there are only a few studies on thermal monitoring. Between these, there are some studies of Serio et al. [12,13,14] that demonstrate how the absolute temperature is affected by environmental conditions and it is influenced by experimental set-up adopted for the tests. Subsequently, it cannot be used as a representative parameter of the FSW process. In particular, a more sensitive thermal parameter is proposed for the monitoring of the FSW process, representing the heat generated during the process. This research has identified a process parameters window suitable for obtaining good quality joints on AA5754 H111. It was shown that thermographic techniques can be effective instruments of control for the FSW process.

In this context, the control and optimization of manufacturing processes have prompted the interest of many researchers towards the study of new technological tools, since it represents a critical issue for the production engineering. In the case of the FSW process, in addition to the use of thermographic techniques, to better understand the process-related dynamics and to control all significant variables it is necessary more than experimental trials. Therefore, it requires the integration of informative technology for enhancing the quality of manufacturing systems. The implementation of numerical and analytical models can reduce time and cost for experiment and analysis through quantitative solutions.

A model based on the adoption of one or more Artificial Neural Networks (ANNs) can help to identify the relation between process parameters and quality of weld. In particular, according to Facchini et al. [15], one of the main advantages of this technique is that it can produce good results, even when supplied data are noisy or incomplete. In these cases, ANN can predict the output parameters after learning from a training data set, where the learning algorithm determines the numeric weights to the link among neurons that produce a robust and correct output. Recently it is spreading the use of ANN to model various problems in many fields such as materials science and the engineering [16,17,18,19,20,21,22]. ANNs are inspired by natural neural networks so they are systems able to process information and simulate the behavior of the brains mechanism.

In summary, the main advantages of a neural network are the ability to implicitly detect complex nonlinear relationships between dependent and independent variables; and the ability to detect all possible interactions between predictor variables and availability of multiple training algorithms.

Neural Networks software packages are very common among scientists and manufacturing researchers. In particular, their applications in the field of welding have showed good success. In scientific literature, an ANN was adopted in order to predict the mechanical properties of butt welding [23]. Yilmaz et al. [24] developed a generalized regression neural network model that allows predicting the tensile strength for steel wires and it was very useful to note that the predicted and the experimental values were very similar. Ates et al. [25] introduced a new technique based on ANNs for the prediction of gas metal arc welding parameters. Input parameters of the model consisted of gas mixtures, whereas the output response of the ANN model included several mechanical properties, i.e., tensile strength, elongation and weld metal hardness. ANN controller was trained with the extended delta-bar-delta learning algorithm. The results showed that the calculated results were coherent with the measured data.

As far as concern the FSW process, in scientific literature, there are only few papers that discuss the modeling of this welding process by a neural network [23,26,27,28,29]. In particular, the most interesting works are those of Shojaeefard et al. [30] based on the adoption of the neural network trained with Particle Swarm Optimization (PSO) for the modeling and the forecast of the mechanical properties of the friction stir welding butt joints in AA7075/AA5083. Asadi et al. [31], adopting the ANN, identified a relationship between the grain size and the hardness of nanocomposites in FSW process.

Concerning the adoption of ANN to the FSW process in case of welding of 5xxx aluminum alloys, the available scientific works are very few. This type of aluminum alloy is widely used in the marine, automotive and aviation fields, thanks to its high resistance to corrosion. In this paper, the design of the ANN was adopted in order to develop a suitable simulation model for predicting, monitoring and controlling the mechanical properties of welded aluminum alloy plates on the basis of FSW process parameters. The data set adopted for training, testing and validation of the ANN, were the results obtained by experimental cases.

The remaining part of this paper is organized as follows: Section 2 presents the experimental procedures; Section 3 presents the simulation model implementation; and Section 4 presents the results analysis and conclusions.

## 2. Materials and Methods

### 2.1. Data Analysis

The present work uses data from previous studies carried out by the FSW process on the alloy AA5754 H111 [12,14,32], in which a qualitative analysis of welded joints with non-destructive (visual inspection) and destructive testing (macrographic tests) was performed in order to detect macro defects present on the surface and within the welded area. The results of the tensile tests of every welded specimens, in terms of ultimate tensile strength (UTS) and Vickers micro hardness, were considered to perform a quantitative analysis of process. Thermal behavior of FSW process was studied through thermographic technique. In particular, two thermal parameters were considered: the maximum temperature and the slope of the heating curve measured during the FSW process (MSHC_RS_ and MSHC_AS_, respectively).

The results obtained showed that the data derived from the thermographic controls can be linked to the quality of welded joints, in terms of UTS. These studies underline how the use of infrared technology for monitoring the FSW process in a quantitative manner, giving important data on the thermal behavior of joints during the process.

**Figure 2:**
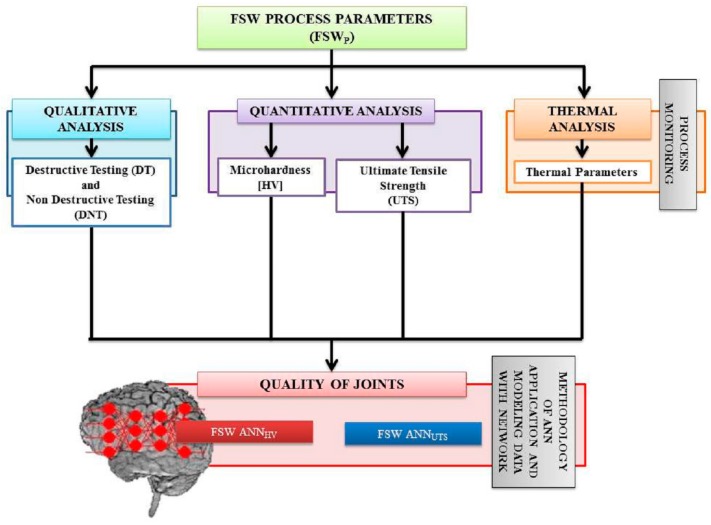
Approaches used for evaluating the quality of FSW process through destructive and non-destructive tests.

Finally, the quality of welded joints, evaluated in terms of UTS and micro hardness, was directly connected to the thermal parameters with the use of the ANN, which is the focus of this work. The model tracked with the use of the neural networks allows predicting quantitatively the mechanical behavior of the FSW joints, as shown in Figure 2.

### 2.2. Collecting the Experimental Data

The used data refer to the experiments on two hot rolled AA5754 H111 plates (6 mm × 200 mm × 200 mm) which were welded with the FSW Process in butt configuration. The AA5754 H111 is not a heat-treatable aluminum-magnesium alloy with nominal Mg content in the range of 2.6%–3.6%. It is supplied in a state of tempers which gives maximum formability to the material. This aluminum alloy is characterized by excellent resistance to corrosion in a marine environment, it has high formability and it is weld able by fusion. Due to its characteristics, it is used to make pressure vessels, components of trucks and tankers, details of chemical plants and in shipbuilding. The nominal composition of AA5754 H111, as registered with the Aluminum Association [33] and physical properties of the material are shown in Table 1 and Table 2, respectively.

**Figure 3:**
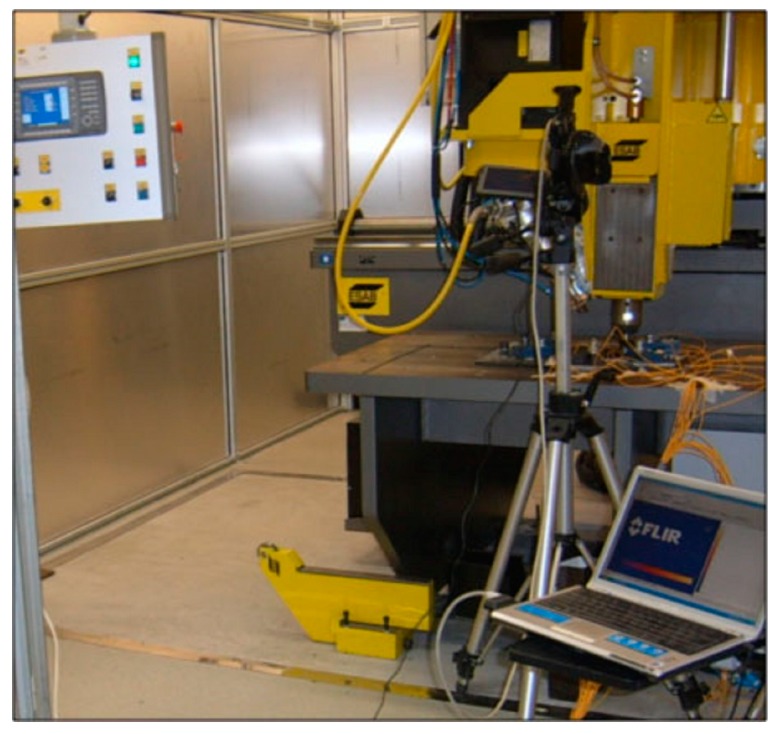
ESAB welding station used for FSW tests.

All the welding tests were carried out in position control, on a Friction Stir Welding Machine-model: LEGIO™ FSW 4UT (for 2-D welding: *Y*-axis range 400 mm; *X*-axis range 1000 mm and *Z*-axis 300 mm), provided by ESAB AB Welding Automation and placed in the TISMA Laboratory (Innovative Techniques for Welding of Advanced Materials, http://www.poliba.it/it/TISMA) of the Department of Mechanical Engineering, Mathematics and Management of the Polytechnic of Bari—as shown in Figure 3.

**Figure 4:**
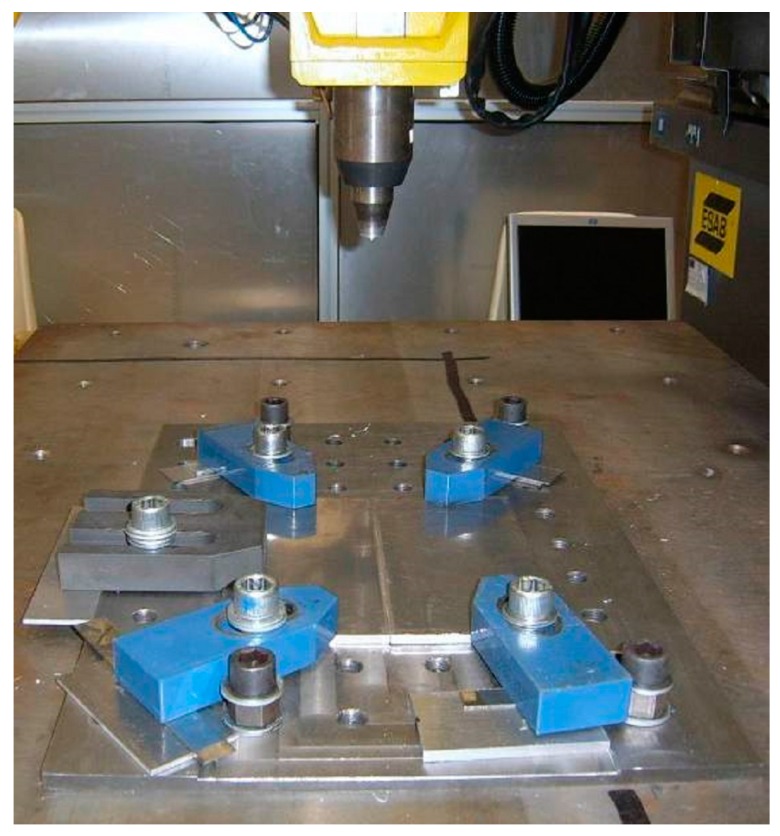
Positioning of the workpiece on the fixture table.

The workpiece was fixed on a rigid backing-plate and clamped along the welding direction on both sides to avoid lateral movement during welding. The terminal part of the workpiece was positioned on the worktables as shown in Figure 4.

The welding direction was parallel to the rolling direction and the dwell time, which is the period needed to preheated the material and to achieve a sufficient temperature ahead of the tool to allow the traverse, was always kept at 15 s, whereupon the tool moved with constant travel speed according to the parameters combination selected and described below. During the penetration phase, the rotating tool pin penetrates into the workpiece until the tool shoulder makes contact. The penetration speed was about 0.5 cm/min. The diameter of the shoulder was 22 mm wide and the tool was inclined at 1.2° with respect to the workpiece to facilitate mixing of the material.

The welding was carried out using the following values of the tool rotation speed and travel speed, which were, respectively, 500–700 rpm and 20–30 cm/min. Using these welding parameters, different samples were welded for destructive and non-destructive testing in order to detect macro defects placed on surface and within the welded area.

Visual inspections and metallographic tests were the first performed examinations. They were carried out preparing the cross-sectional samples taken from all welded joints. Specimens were prepared using standard metallographic methods for macroscopic examinations of the weld zones. Cross sections of the welds were cold mounted, polished and etched with a solution consisted of 5 mL of distilled water and 120 mL of hydrochloric acid for 90 s. After these treatments, the bead appearance was observed by capturing the images detected from the cross sections to identify large and very small internal flaws. The surface appearance of FSW showed a regular series of partially circular ripples. These ripples were essentially cycloidal and were produced by the circumferential edge of the shoulder during traverse. Many tests have showed continuous flash but with a marked ripple. This has demonstrated the significant ductility of the material and that the plastic deformation suffered by the material changes periodically over time. Visually the welds carried out with the greater rotation speed were unwrapped, because they have a macro voids in the section, denominated “tunnel”. These defects were caused by the use of incorrect process parameters, which provided wrong heat input. It produced a wrong mixing action of the material and it created voids in the section and on the surface of the welding.

Macrographic analyses were carried out to detect internal flaws of the welds. Almost all of the analyses revealed a good mixing and a good penetration of the tool in the joints, except for the joints section realized using the highest rotation speed (n = 700 rpm) where defects were observed such as cavity, due to non-appropriate contribution of heat input and stirring rate.

All macrographs presented a nugget shape, with a relevant elongated form and the typical “onion rings”, identifying by the mixing zone characteristic of FSW process, were very visible. The combination of speeds, 500 rpm and 20 cm/min, exhibits a high quality with no significant defects.

Transverse tensile tests, at room temperature, have done to evaluate the mechanical properties of welded joints. All tests were performed on a MTS servo hydraulic machine (Model 370, MTS, Eden Prairie, MN, USA), under displacement control with a constant crosshead speed displacement rate of 5 mm/min according to Standard UNI EN ISO 6892-1:2009 [34].

Specimens were cut distant to the start position and the exit the tool because, around these positions, the weld process was not stationary. In particular, they were machined and prepared according to standards UNI EN ISO 6892-1:2009 and UNI EN ISO 25239:2011 [34,35] and they were obtained in orthogonal direction with respect to the rolling direction. The gauge section of specimens was located within the welded zone and the geometrical dimensions chosen were 12 mm width and 200 mm length for a gauge length of 50 mm. The results of the tensile tests showed that the maximum values of UTS were achieved for a tool rotation speed of 500 rpm, and a travel speed of 20 cm/min.

Documented statistical analysis [14,32] point out the dependence of the mechanical characteristics of the AA5754 H111 FSW joints in terms of UTS from the tool rotation speed and the position along the welding direction.

**Figure 5:**
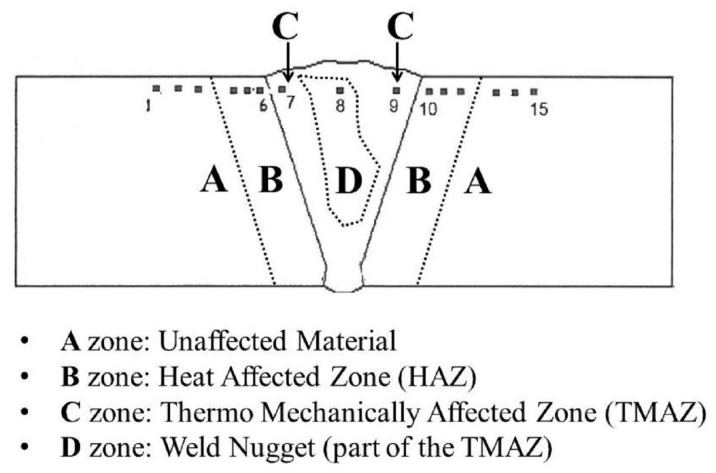
Points for the measurement of Vickers micro hardness for the FSW joint.

Micro hardness tests were conducted considering changes in the microstructure of the FSW joints which is the result of the welding process. The FSW process was asymmetric and the thermo-mechanical action, due to movement of the tool with the surface of the workpiece, creates a microstructure evolution in the welded zone. Therefore, moving the FSW joint outward, the following areas were observed, as shown in Figure 5: the nugget zone, which is the part invested by the pin, the TMAZ (Thermo-Mechanically Affected Zone) and, finally, the HAZ (Heat Affected Zone) and the base material. The geometry of the nugget can be changed by varying the speed of rotation of the tool.

The effects of the FSW on the hardness distribution were fully analyzed. It was observed that the weld samples with the same travel speed had similar profiles. From the results, no HAZ softening was found which was expected because the tempered H111 is almost equivalent to the O-temper. In almost all of tests, the highest value of micro hardness in the stirred zone was found not in the middle of the joint but shifted towards one of the sides of the joint, where higher plastic strain was observed and the micro hardness curve shows a W-shape. The average hardness, in the nugget and in the base material, was similar among all the samples. The hardness profile greatly depends on the precipitate distribution and only slightly on the grain and dislocation structure [36]. Therefore, the evolution of the precipitate distribution, with the experienced temperature peak and with the stain introduced during the welding, produces the observed hardness variation. The analysis of all samples, conducted with different process parameters, show different values of the mechanical properties; the ultimate tensile strength (UTS) and the HAZ micro hardness of all the FSW joints were used to perform a quantitative analysis of process and to describe the influence of process parameters on the quality of joints (Table 3).

In addition, the thermal behavior of FSW process was investigated through thermographic technique. A detailed discussion about these results is clearly present in the work of Serio et al. [13]. In particular, for each test, the slope of the heating curve measured during the FSW process was evaluated. This parameter allowed evaluating some important thermal characteristics of the joints, such as the percentage of heat induced by the joint and its relative materials speed of heating. The control of the slope of the heating curve has allowed us to monitor the FSW process in quantitative way revealing the thermal behavior of joints during the process. The main considerations about the thermal behavior of joints can be summed up as follows:

- The higher temperatures were measured along the retreating side for all tests.
- The maximum temperature reached during the process, pixel by pixel, can be used to monitor the stationary nature of the process.
- The Maximum Slope of Heating Curve (MSHC) of thermal profiles evaluated on the surface of joints can be used for monitoring the process parameters. This parameter is more sensitive than the maximum temperature as it is directly correlated with the energy and then the heat supplied during the welding process.

In addition, in this case, statistical analyses were carried out in order to verify the statistical significance of the effect produced by process parameters on MSHC [14]. The analysis of variance ANOVA showed that the parameter MSHC was influenced by the tool rotation speed, the travel speed and position.

The results of all tests are summarized in the Table 3 and the same data were used to train the ANN.

## 3. ANN Simulation Model

### 3.1. Design and Training of the ANNs

The simulation model was developed in order to establish a relationship between the mechanical properties of the joints and the technical parameters of the FSW process. Two different ANNs were adopted; the first network (ANN_HV_) was used for identify the Vickers micro hardness of HAZ on the bases of five different input parameters (n, v, p, MSHC_RS_, and MSHC_AS_); the second network (ANN_UTS_) considers the Ultimate Tensile Strength. The inputs of the ANN_UTS_ were 6-component vectors, five of them were the same of the first network (n, v, p, MSHC_RS_, and MSHC_AS_), the sixth input parameter, was HV_haz_ (the value estimated by first network). The ANNs were implemented using Alyuda NeuroIntelligence™-Neural networks software (2.2, Alyuda Research Company, LLC., Cupertino, CA, USA). At the beginning, all data are preprocessed to simply convert the input data into a new version for three reasons [37]: (i) to ensure the size of data reflect the importance level in determining the output; (ii) to facilitate the random initialization of weights before training the networks; and (iii) to normalized all data to avoid different measurement due to different unit of input. The configurations adopted for learning process are as following: logistic function was selected as activations function for all neurons, the learning and momentum rates was fixed at 0.01, and the training process stopped when the model’s mean squared error reduces by less than 1 × 10^−6^ or the model completes 2.5 × 10^5^ iterations, whichever condition occurs first.

In order to identify the best architecture, all training algorithms supported by Alyuda NeuroIntelligence™ were applied to the analysis of the data. The training algorithms were: Quick Propagation (QP), Conjugate Gradient Descent (CGD), Quasi-Newton (Q-N), Limited Memory Quasi-Newton (LMQ-N), Levenberg-Marquardt (L-M), Online Back Propagation (OBP), and Batch Back Propagation (BBP). The performance of each network was evaluated adopting both the Correlation Coefficient, r [−1,1] and the Coefficient of Determination, R^2^ [−1,1]. It is convenient to note that R^2^ is equal to r in linear regression analyses, but that is not necessarily the case in ANN [38].

In most cases, the optimal R^2^ and r was obtained for batch back propagation (BBP) learning algorithm (Table 4), therefore both ANNs were trained with BBP training algorithm based on a recursive procedure that estimates the weights according to the response of each layer errors [36,39].

The synaptic weights were updated using the method of gradient descent to minimize error (which evaluates the difference between the expected value (target) and real value of the measure (see Equation (1))).

*e* = *x* − *d*, (1)

According to the neural network routine, the transformation of the input vector into output vector was identified as function *f* (see Equation (2)), where “*wih_ij_*” value identifies the weight attributed to the connection from node *i* of the input layer to the node j of the hidden layer, while “*who_ij_*” represents the value of the weight which underlined the link between hidden land output layer:

*y*_0_ = *f* (Σ*_j_* (*f* (Σ*_i_wih_ij_* × *x_i_*) × *who_ij_*)), (2)

The learning process was characterized by iterative system finalized to the identification of the optimal combination of the matrix w*, through the minimization of *E*(*w*) function (see Equation (3)).

*E*(*w*) = (1/2) × Σ*_p_* Σ*_i_* (*x_i_^p^* − *d_i_^p^*)^2^, (3)

where *x* is the result produced by the matrix of weights w*, and d is the “desired” product.

### 3.2. ANN_HV_ Prediction Model

The first network ANN_HV_ was developed with five input nodes (n, v, p, MSHC_RS_, and MSHC_AS_) and only one response node (output node), identified as the micro hardness of the Heat Affected Zone of the welds, HV_haz_. The data sets are displayed in Table 3 and all outputs were normalized in the range 0–1 to improve the stability of the neural network. In this case, in order to reduce the overfitting phenomenon and avoid a excessive compute time, the available data set (made up by small sample size) was partitioned into three subsets adopting the Leave-one-out cross-validation (LOOCV) procedure; consequently, three different subsets were identified.

- Training set: The group of data constituted by a sample of 75% of total data for training the ANN. The synaptic weights were, in this phase, repeatedly updated in order to reduce the error between experimental outputs and respective targets;
- set: This group of data includes a sample of 12.5% of total data, given to the network during the learning phase, in this one the error was evaluated in order to update the threshold values and the synaptic weights;
- set: This group of data includes a sample of 12.5% of total data. This phase consists of identifying the underlying trend of the training data subset, avoiding the overfitting phenomenon. In the case of the error measured, compared to validation subset, begins to increase, the training was stopped. This procedure runs together with the training procedure.

**Figure 6:**
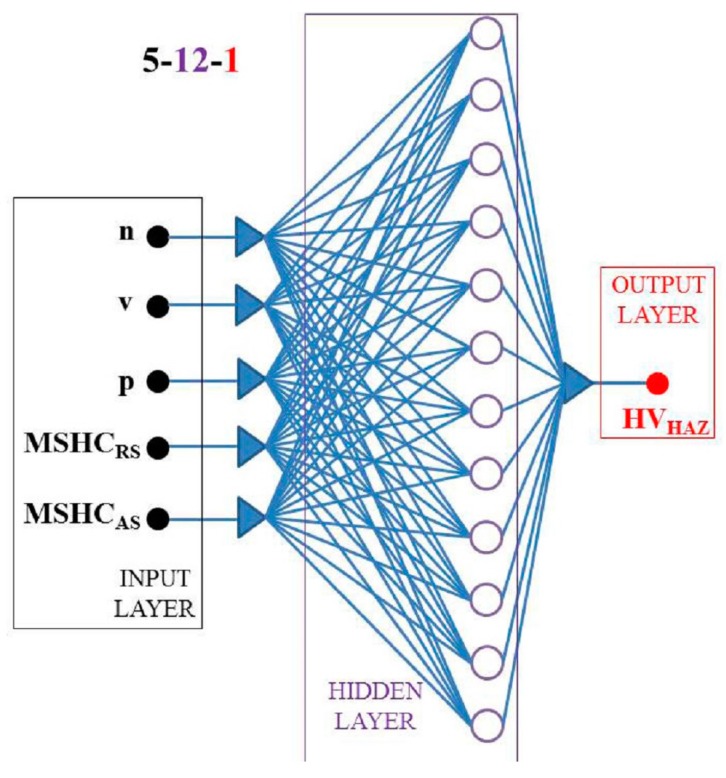
Back-propagation neural network used to foresee the Vickers micro hardness of the Heat Affected Zone (HAZ).

**Figure 7:**
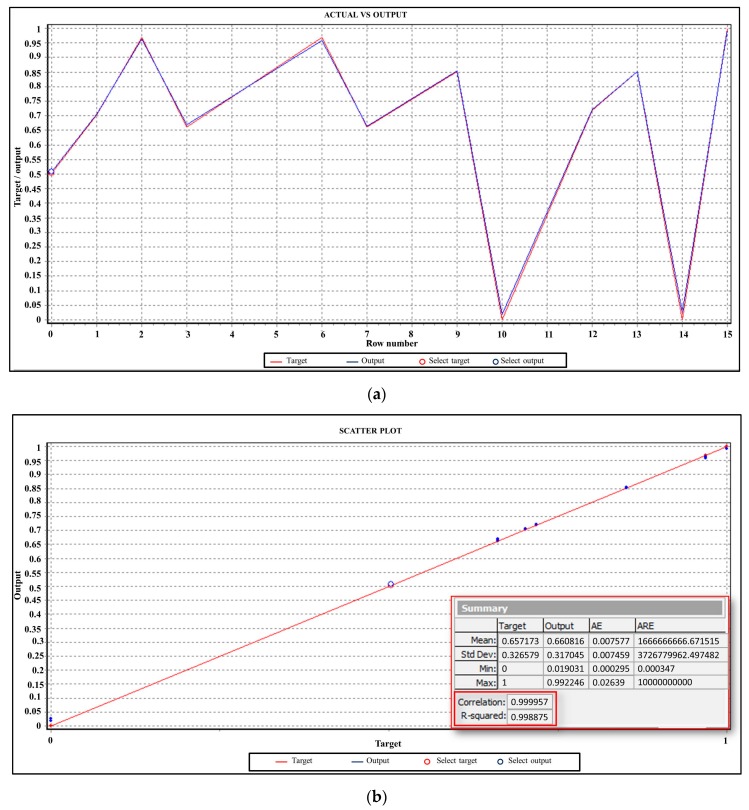
Simulation results: (**a**) Predicted Vickers HAZ micro hardness by ANN versus the experimental data; and (**b**) regression line at the training stage.

To identify the “best” architecture of the network, it was adopted a “trial and error approach”: that it considered more of 1000 different networks architectures. The network fitness score, based on the inverse of the mean absolute error (MAE) on the testing set, was computed by software for every network with different design (number of hidden layers, number of nodes, etc.). A higher fitness score allowed identifying the better network architecture. Consistently, for each configuration, the minimum prediction error was evaluated and the best accuracy was achieved adopting an ANN_HV_ characterized by only one hidden layer with 12 neurons as shown in Figure 6 and Figure 7.

### 3.3. ANN_UTS_ Prediction Model

**Figure 8:**
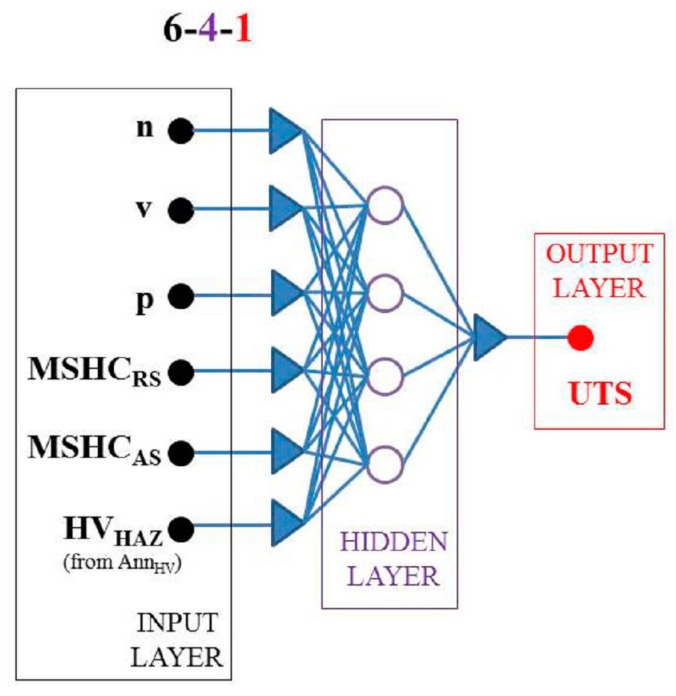
Back-propagation neural network used to foresee the Ultimate Tensile Strength.

**Figure 9:**
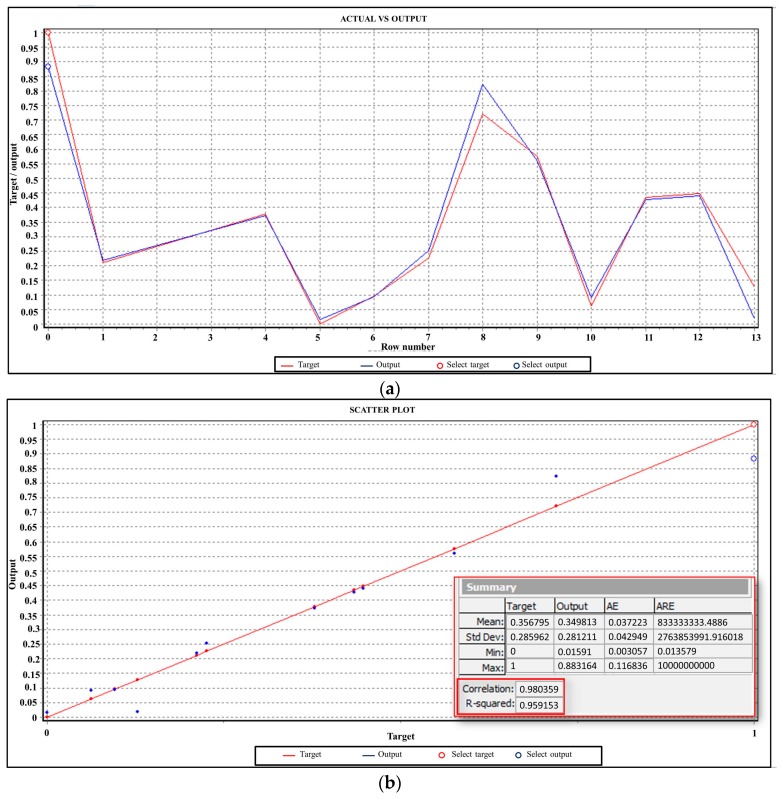
Simulation results: (**a**) Predicted Ultimate Tensile Strength by ANN versus the experimental data; and (**b**) regression line at the training stage.

The second network (ANN_UTS_) was developed with six input nodes (n, v, p, MSHC_RS_, MSHC_AS_, and HV_haz_) and one response node (output) identified as the Ultimate Tensile Strength of the welds (UTS). In this case, the criteria adopted for dataset partition, the samples size of the three subsets (training, testing, and validation) and the methodology chosen for identifying the architecture of the network are the same of the ANNHV (see Section 3.2). The “best” reliability of the prediction was achieved, adopting an ANN_UTS_ characterized by only one hidden layer with four neurons, as shown in Figure 8 and Figure 9.

## 4. Results and Discussion

### 4.1. Experimental Results

**Figure 10:**
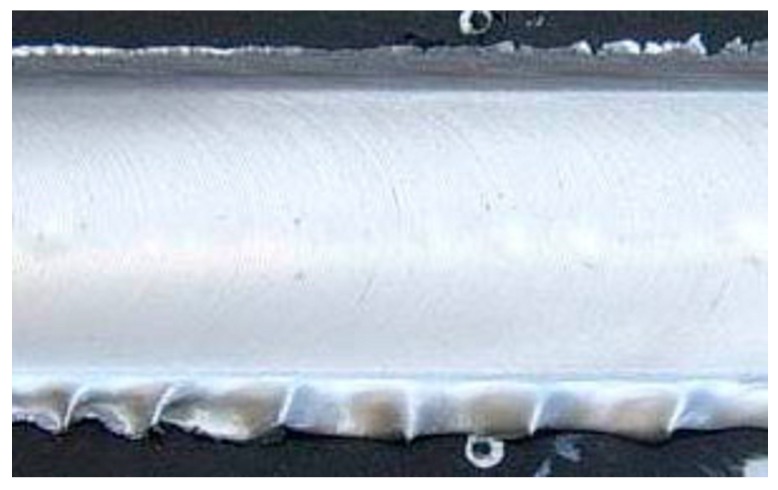
Surface of the joint which verified the best results in mechanical terms (n = 500 rpm; v = 20 cm/min).

**Figure 11:**
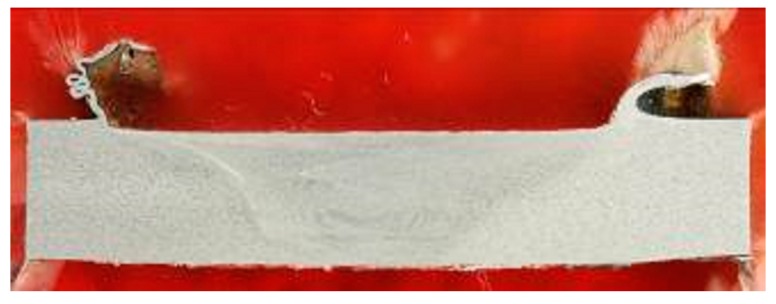
Cross section of the joint which verified the best results in mechanical terms (n = 500 rpm; v = 20 cm/min).

Figure 10 and Figure 11 illustrate the characteristics of the surface and in section of the welded sample, which showed the best results in mechanical terms. The pair of process parameters, n = 500 rpm and v = 20 cm/min, led to perform welding with high mechanical characteristics and high quality with no significant defects both on the surface and in the section.

Moreover, based on the reference weld pitch (WP = 0.35 mm), which indicated the ratio between the travel speed v and the tool rotation speed n [2], the sample welded with n = 500 rpm and v = 20 cm/min was the one nearest to the optimal ratio (WP = 0.40 mm).

**Figure 12:**
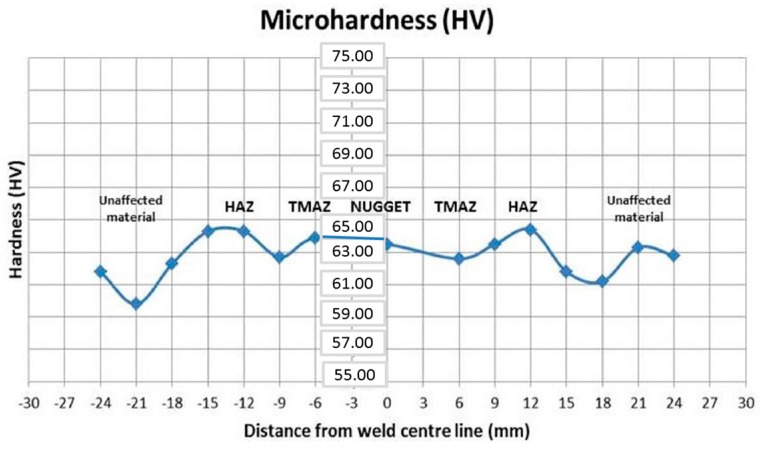
Micro hardness distribution of a significant test.

In almost all of the tests, the highest value of micro hardness in the stirred zone was found not in the middle of the joint, but shifted towards one of the sides of the joint, where higher plastic strain was observed and the micro hardness curve shows a W-shape, as shown in Figure 12. The average hardness in the nugget and in the base material was similar among all the samples.

However, the results obtained from the experiments [32] motivate an optimization of technological parameters in order to increasing the seam quality, since it promotes a stable and defects-free process.

### 4.2. Model Validation

In order to evaluate the reliability of the mechanical properties predicting by simulation model, the features of the weld bead in output by ANN was compared to experimental results.

For this purpose, the Mean Absolute Percentage Error (MAPE) was calculated for the two ANNs modeled. The results obtained from the use of models (Table 5) demonstrated that the predicted parameters ensure a higher level of reliability. Therefore, the neural networks were able to predict, with significant accuracy, the mechanical properties of the friction stir welding joints, under a given set of welding conditions.

Since the results achieved in the experiments have shown the most significant process parameters for the friction stir welding process applied on AA5754 H111 plates [32], the development of this ANN model could be used to identify the optimal process parameters setting in order to achieve the desired welding quality.

## 5. Conclusions

The results showed that the simulation model could be used as an alternative way for predicting, controlling and monitoring the FSW process. The MAPE obtained for the outputs micro hardness (HV_HAZ_) and ultimate tensile strength (UTS) were, respectively, 0.29% and 9.57%. The R^2^ values were in all cases very high, in particular, R^2^ values corresponding to 0.94 for the prediction of HAZ parameter and 0.96 in case of the UTS forecast were observed. Although the prediction of UTS was characterized by higher level of MAPE if compared to HAZ estimated value, it was, however, considered acceptable under technological perspective. According to a prudential approach, it is possible to overstate the prediction of UTS of the 10%, in this way a high reliability of the model for many industrial environments will be ensured.

The adoption of the simulation model can be very useful for the friction stir welding process. Nowadays, for this kind of process and materials, in the scientific literature, there are no models able to predict the mechanical parameters on the basis of weld process parameters. The approach suggested in this paper allows identifying one or more unknown parameters starting from experimental data, and the reliability of the forecasting model increases with increasing of the amount of available data. This means that as time passes, upgrading the model with new data (this is possible by means of a user friendly ANN-interface), the prediction model improves the reliability of the forecasting and will allow optimizing the quality of the weld joints through the identification and the control of process parameters.

In many cases, the reduction of weld defects translates into greater safety and in a decrease of the repairs, providing predict a drastic reduction of the costs related to additional testing that can be replaced thanks to the estimation of the mechanical properties of the joints.

In this case, a small sample size for the prediction of the target parameters was adopted, and the forecasts of HV_HAZ_ and UTS were considered to be acceptable under technological perspective. In order to improve the reliability of the model, it is necessary, therefore, to adopt an increased number of experimental data for training, validation and test phase. Future research, moreover, could be conducted adopting other software packages, which are able to provide the internal process of learning which describe the reasoning behind a prediction provided by neural networks, as, in this case, Alyuda software could not provide it.

Finally, this work suggests that the full integration of analysis, prediction, control and continuous learning into a single framework is promising, not only in friction stir welding process but also in the prospect of other manufacturing technologies.

## Figures and Tables

**Table 1:** Alloying elements and their nominal weight percentage in AA5754 H111.

Mg	Mn	Fe	Si	Cr	Zn	Ti	Cu
2.60–3.60	0.50	0.40	0.40	0.30	0.20	0.15	0.10

**Table 2:** Physical properties of AA5754 H111.

Thermal Conductivity (W/m°C)	Specific Heat (Cal/kg°C)	Density (g/cm^3^)	E (MPa)	HBS	Rm (MPa)	Rp (0.2) (MPa)
147 at 20 °C	0.213 at 20 °C	2.66 at 20 °C	70,000	63	190	80

**Table 3:** Measured data used to train the ANN.

INPUT					OUTPUT			
n (rpm)	v (cm/min)	p (mm)	MSHC_RS_ (°)	MSHC_AS_ (°)	HV_haz_	HV_haz norm._	UTS (MPa)	UTS_norm._ (MPa)
20	500	20	86.05	85.83	60.88	0.50	166.69	1.00
30	700	20	87.37	87.37	61.93	0.70	70.25	0.21
20	700	20	87.12	87.92	63.33	0.97	120.75	0.62
30	500	20	86.88	86.85	61.72	0.66	80.05	0.29
20	500	120	87.25	86.80	60.88	0.50	90.66	0.38
30	700	120	88.14	88.15	61.93	0.70	44.29	0.00
20	700	120	87.23	87.91	63.33	0.97	56.06	0.10
30	500	120	87.97	87.91	61.72	0.66	71.99	0.23
20	500	20	86.60	86.16	62.02	0.72	132.43	0.72
30	700	20	87.74	88.23	62.72	0.85	114.87	0.58
20	700	20	86.53	88.00	58.23	0.00	51.86	0.06
30	500	20	89.07	87.23	63.50	1.00	97.43	0.43
20	500	120	87.59	87.43	62.02	0.72	99.06	0.45
30	700	120	88.53	88.32	62.72	0.85	59.95	0.13
20	700	120	87.48	87.74	58.23	0.00	46.55	0.02
30	500	120	87.58	88.45	63.50	1.00	113.98	0.57

**Table 4:** Performance of the ANN adopting different learning algorithms.

Target	Training Algorithm	Correlation Coefficient (r)			Coefficient of Determination (R^2^)		
		Train.	Valid.	Test.	Train.	Valid.	Test.
HV_HAZ_	QP	0.89	0.91	0.85	0.88	0.84	0.81
HV_HAZ_	CGD	0.79	0.87	0.86	0.77	0.86	0.75
HV_HAZ_	Q-N	0.78	0.56	0.61	0.66	0.49	0.46
HV_HAZ_	LMQ-N	0.83	0.88	0.78	0.76	0.85	0.64
HV_HAZ_	L-M	0.76	0.68	0.78	0.54	0.52	0.51
HV_HAZ_	OBP	0.89	0.92	0.95	0.84	0.86	0.91
HV_HAZ_	BBP	0.96	0.97	0.94	0.94	0.97	0.93
UTS	QP	0.78	0.85	0.76	0.74	0.79	0.65
UTS	CGD	0.56	0.45	0.39	0.39	0.31	0.18
UTS	Q-N	0.45	0.54	0.25	0.32	0.28	0.21
UTS	LMQ-N	0.66	0.69	0.49	0.59	0.66	0.46
UTS	L-M	0.68	0.75	0.45	0.58	0.57	0.39
UTS	OBP	0.95	0.95	0.96	0.91	0.89	0.88
UTS	BBP	0.98	0.98	0.99	0.97	0.96	0.94

**Table 5:** Mean absolute percentage error computed for hardness and ultimate tensile strength of AA AA5754 H111 plates welded with the friction stir welding process.

ANN Model	Input Parameters	Output Parameters	MAPE (%)
ANN_HV_	n	HV_haz_	0.29
	v		
	p		
	MSHC_AS_		
	MSHC_RS_		
ANN_UTS (in cascade)_	n	UTS	9.57
	v		
	p		
	MSHC_AS_		
	MSHC_RS_		
	HV_haz_ (predicted with the ANN_HV_ model)		

## Abbreviations

The following abbreviations are used in this manuscript:

FSW	Friction Stir Welding
AA	Aluminum Alloy
UTS	Ultimate Tensile Strength
IRT	Infrared Thermography
HAZ	Heat Affected Zone
TMAZ	Thermo-mechanically affected
MSHC	Maximum Heating Slope
HV	Vickers Hardness
ANN	Artificial Neural Network
WP	Weld Pitch
